# Detecting single-nucleotide polymorphism by single-nucleotide polymorphism interactions in rheumatoid arthritis using a two-step approach with machine learning and a Bayesian threshold least absolute shrinkage and selection operator (LASSO) model

**DOI:** 10.1186/1753-6561-3-s7-s63

**Published:** 2009-12-15

**Authors:** Oscar González-Recio, Evangelina López de Maturana, Andrés T Vega, Corinne D Engelman, Karl W Broman

**Affiliations:** 1 Department of Dairy Science, University of Wisconsin-Madison, 266 Animal Science Building, 1675 Observatory Drive, Madison, Wisconsin 53706, USA; 2 Department of Animal Sciences, University of Wisconsin-Madison, 1675 Observatory Drive, Madison, Wisconsin 53706, USA; 3 Consejería de Sanidad, Observatorio de Salud Pública, Valladolid 47007, Spain; 4 Department of Population Health Sciences, University of Wisconsin-Madison, 707 WARF Building, 610 North Walnut Street Madison, Wisconsin 53726, USA; 5 Department of Biostatistics and Medical Informatics, University of Wisconsin-Madison, 600 Highland Avenue, Madison, Wisconsin 53792, USA

## Abstract

The objective of this study was to detect interactions between relevant single-nucleotide polymorphisms (SNPs) associated with rheumatoid arthritis (RA). Data from Problem 1 of the Genetic Analysis Workshop 16 were used. These data consisted of 868 cases and 1,194 controls genotyped with the 500 k Illumina chip. First, machine learning methods were applied for preselecting SNPs. One hundred SNPs outside the HLA region and 1,500 SNPs in the HLA region were preselected using information-gain theory. The software *weka *was used to reduce colinearity and redundancy in the HLA region, resulting in a subset of 6 SNPs out of 1,500. In a second step, a parametric approach to account for interactions between SNPs in the HLA region, as well as HLA-nonHLA interactions was conducted using a Bayesian threshold least absolute shrinkage and selection operator (LASSO) model incorporating 2,560 covariates. This approach detected some main and interaction effects for SNPs in genes that have previously been associated with RA (e.g., rs2395175, rs660895, rs10484560, and rs2476601). Further, some other SNPs detected in this study may be considered in candidate gene studies.

## Background

Rheumatoid arthritis (RA) is a chronic disease with known autoimmune pathophysiology. RA is a heritable condition and association studies have already identified a genomic region in chromosome 6 (the HLA region). While this represents progress in elucidating genetic contributions to RA, much is still unknown about the underlying genetic causes, and there is plenty of evidence that there exist other genes affecting disease risk, both as major effects and in epistasis [1]. Genome-wide association studies (GWAS) of diseases and complex traits have become a major focus of research in human genetics. GWAS may provide robust results for acquiring knowledge on the underlying genetic behavior of RA. One of the most difficult challenges of GWAS is how to deal with the large *p*, small *n *problem, arising when the number of variables considered (*p*) is much larger than the number of subjects (*n*). The problem becomes particularly difficult when one seeks to estimate single-nucleotide polymorphism (SNP) by SNP interactions. One approach for efficiently handling high dimensional GWA data consists of two steps: 1) reducing dimensionality by filtering non-informative markers, and 2) applying a more sophisticated model to quantify the effect of the selected SNPs and their interactions. Information gain and the *wrapper *procedure are examples of machine learning that have shown benefits over linear regression for Step 1. These methods are easy to implement and may deal with crude, noisy, and inconsistent information. They alleviate redundancy, colinearity, and the assumption of multivariate normality, making them appealing in genomic studies. The function that relates covariates to observations is unspecified, providing more flexibility in the model. Further, they may deal with non-additive effects, which are of interest in genetic epidemiology. Some drawbacks to these methods exist: information gain may not completely remove colinearity in the system and the *wrapper *procedure it is too computationally demanding to use on a large number of records and SNPs. Hence, a second step is necessary. A large variety of methods have been previously proposed for Step 2. Here, we propose a novel method that is able to reduce colinearity and make a higher shrinkage to zero than other methods for less relevant SNP and SNP × SNP effects.

The Genetic Analysis Workshop 16 (GAW16) provides an opportunity for testing novel methods, such as those proposed above, on a well characterized dataset, to compare results and interpretations, and to discuss current problems in genetic analysis. The aim of this study was to identify additional disease susceptibility loci in the GAW16 RA data in a two-step approach: first, reducing the number of SNPs to be tested through machine learning algorithms; second, identifying interactions or epistatic effects between HLA and non-HLA SNPs using a Bayesian threshold LASSO model.

## Methods

### Data

Data from the North American Rheumatoid Arthritis Consortium (NARAC) provided by the GAW16 were used in the analyses. The initial batch consisted of 868 cases and 1,194 controls genotyped with the 500 k Illumina chip (545,080 SNPs). Further description of the initial data can be found in Plenge et al. [2]. Local institutional review board (IRB) approval was obtained.

#### Quality control

SNPs showing >2% missing genotypes were excluded (64,041). Monomorphic SNPs were omitted (1,920). SNPs with minor allele frequency <0.05 that did not show association with the disease through a Fisher's exact test (*p *< 0.001) were also omitted (47,190). Finally, SNPs that deviated from Hardy-Weinberg equilibrium (*p *< 0.0001) in the controls were discarded (11,835). The number of SNPs remaining in the analysis was 420,094.

### Stage 1: Preselection of significant SNPs using machine learning

#### Information gain or entropy theory

Pre-selection of informative SNPs to be included in Stage 2, and substantially reduce the feature space, was performed using the information gain or entropy reduction criterion [3]. The entropy of the probability distribution of a discrete random variable **y **is defined as:

where *A *is the set of all states that **y **can take, the logarithm is on base 2 to mimic bits of information, and we take 0 log 0 = 0. Here, **y **refers to case and control phenotypes. For each SNP, the data set was divided into three subsets corresponding to the three possible genotypes (aa, Aa, or AA). For each genotype *k*, there are  individuals showing the disease, and  individuals with absence of disease. The information gain for each SNP *s *(*s *= 1, 2,...,*p*) is the difference in entropy of the probability distribution (the reduction of uncertainty) before and after observing genotypes at each SNP, calculated as:

where . At this point, SNPs were divided in two groups based on whether they were in the HLA region or somewhere else along the genome (non-HLA SNPs). The HLA region was defined starting at HLA-F (29,799,096 to 29,803,052) and extending to HLA-DPB1 (33,151,738 to 33,162,954). The 100 non-HLA SNPs with the highest information gain were selected to test for interactions with HLA SNPs in Stage 2. The HLA SNPs that passed to Stage 2 were selected as follows.

#### Selection of independent SNPs in the HLA region on chromosome 6

The most relevant SNPs in this region were selected using the *wrapper *procedure [4]. This procedure aims to reduce redundancy and colinearity in a feature subset. The HLA SNPs with an information gain above the 99.65 percentile (approximately the top 1,500 SNPs) were considered as candidates, and were included in this *wrapper*. This method involves searching through all possible combinations of SNPs in the data to find an 'optimal' subset of SNPs that best classify the phenotype outcome (binary: case or control), using an attribute evaluator and a search method. The attribute evaluator used was the naïve Bayesian classifier, with a bidirectional hill-climbing search method. The naïve Bayesian evaluator can be described as follows:

Given an observed phenotype *y *with genotype *k*_1_,...,*k*_*p*_, the best prediction (classification decision) is given by class value *Y *(case or control) such that Pr(*Y *= *y*|*K*_1 _= *k*_1_, ..., *K*_*p *_= *k*_*p*_) is maximum. Applying Bayes' theorem gives

The prior probability of the phenotype, PR(*Y *= *y*), can be estimated from the training data, and the PR(*K*_1 _= *k*_1_, ..., *K*_*p *_= *k*_*p*_) cancels out when the odds of class membership are calculated. Then, assuming that the genotypes are conditionally independent, the probability of each genotype conditioned to the observed phenotype can be estimated as:

We chose a five-fold cross-validation scenario in which a different list of SNPs was generated in each fold. Therefore, SNPs that appeared in three or more folds were extracted and included in Stage 2. The *wrapper *approach was implemented using the *weka *software [5].

### Stage 2: Selection of significant SNPs and interactions

To identify interactions among SNPs in the HLA region and between an HLA SNP and a SNP elsewhere in the genome, we applied a Bayesian version of the LASSO (least absolute shrinkage and selection operator) [6]. The LASSO constrains the sum of absolute values of the regression coefficients, leading some coefficient estimates to be exactly zero. This can be viewed as a feature selection, and is suitable for quantifying estimates, as well.

The binary nature of the outcome (control vs. case) was taken into account by applying a Bayesian threshold LASSO BTL model, a modification of the Bayesian LASSO [7], the performance of which will be tested for the first time in this study. The traditional threshold model [8] postulates that there is an underlying random variable called liability (*λ*) that follows a continuous distribution, and that the observed dichotomy is a result of the position of the liability with respect to a fixed threshold:

Here, the liability was taken as the response variable. The BTL can be described as:

where ***λ ***is the vector of liabilities for all individuals, ***β ***are the LASSO estimates with their respective incidence matrix **X**, and, as a modeling choice, **e **was considered the vector of residuals independent and identically distributed as . In accordance with tradition, we fixed the threshold to be 0 and the residual variance to be 1; alternate choices result in the same model.

Let **Xβ **= **X**_*M*_***β***_*M *_+ **X**_*HLA*-*HLA *_***β***_*HLA*-*HLA *_+ **X**_*HLA*-*nonHLA *_***β***_*HLA*-*nonHLA*_, where ***β***_*M *_is the vector of major effects, ***β***_*HLA*-*HLA *_corresponds to the vector for interaction effects between HLA SNPs, and ***β***_*HLA*-*nonHLA *_is to the vector for interaction effects between HLA and non-HLA SNPs, with **X**_*M*_, **X**_*HLA*-*HLA*_, and **X**_*HLA*-*nonHLA *_being the corresponding incidence matrices. These incidences matrices were constructed such that each major effect for SNP *j *was codified as  = (-1, 0, or 1) for aa, Aa, and AA, respectively. Interactions were codified as follows: for each *SNP*_*j *_we defined two covariates,  and , with  = 1if the genotype was coded as 0 (0, otherwise), and  = 1 if the genotype was coded as 1 (0, otherwise). Then, for each *SNP*_*j *_× *SNP*_*k *_interaction, we set four covariates,  for *m*, *n *equal to 1 or 2.

Eq. (1) for individual *i *can be written as:

In a fully Bayesian context, the LASSO estimates (**β**) can be interpreted as posterior modes estimates when the regression parameters have independent and identical double-exponential priors [6]. Park and Casella [7] proposed using a conditional Laplace prior specification for the LASSO estimates of the form:

Samples from posterior distributions of those estimates were drawn from the Gibbs sampling algorithm described in Park and Casella [7], with a chain length of 100,000 samples discarding the first 50,000 as burning, after checking convergence.

## Results

Information gain was calculated for each SNP across all chromosomes. The total entropy of the data was 0.98, which is the maximum information gain that a feature (i.e., SNP) could provide. The highest information gain was found in the HLA region on chromosome 6, as expected, with a maximum value of 0.19 for SNP rs2395175. Thirteen other SNPs in this region had an information gain higher than 0.10. The highest information gain outside of the HLA region was 0.017 (rs2476601 on chromosome 1). Within the HLA region, 472 out of the 1,323 SNPs had information gain in the 99.65 percentile. The wrapper procedure selected 6 out of the 472 SNPs.

**Figure 1:**
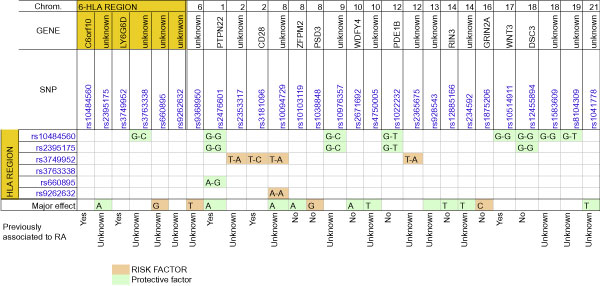
**Major effects and interaction basis functions detected by the Bayesian threshold LASSO model**. Allele or interaction alleles are specified. The allele for the HLA SNPs is specified first in the interactions.

Therefore, 2,560 covariates (100 main effects, 4 ×  HLA-HLA interactions, and 4 × 6 × 100 HLA-non HLA interactions) were introduced in the BTL model. As expected, the posterior means of a large amount of effects were shrunk to zero. The main effects or epistatic basis functions with at least 80% of the posterior distribution either higher or lower than zero, are shown in Figure 1. Among those, the LASSO included the main effects of the two HLA SNPs with the highest information gain (rs2395175 and rs660895). The interaction with the largest effect was that between SNPs rs10484560 (HLA region) and rs2476601 (chromosome 1). These SNPs belong to genes that were previously reported as part of one of the most important interactions for RA [9]. Further, 4 out of the 21 non-HLA SNP shown in Figure 1 were in genes that had been previously related to RA, such as rs3181096 or rs10514911 [10,11].

## Conclusion

A pre-screening stage seems necessary in genome-wide association studies to reduce the large *p*, small *n *problem. The machine learning approach (information gain + *wrapper*) used in this study detected the most important known region associated with RA and reduced the number of SNPs in both the HLA region and across the genome. In a second stage, the BTL model selected covariates of major and epistatic effects strongly associated with RA, some of them already known. The major effects of the two HLA SNPs with highest information gain did appear in the top 27 covariates, showing their importance on the liability to RA.

Shi et al. [12] used a LASSO model on the simulated data from the GAW15 Problem 3. However, they used a different pre-screening method and a non-Bayesian version of the LASSO. The Bayesian counterpart provides a measure of the reliability of the estimates. Because data used in the GAW16 RA problem are real data, the accuracy of the proposed approach cannot be tested immediately. However, the results in this study can be compared to the results generated by other methods applied to the same data, and also with past and future analyses. Further, SNPs found in this study with unknown previous function might act as markers of candidate genes in future research. Proving the benefits of these methods over others widely used in the field is a challenge for the future.

## List of abbreviations used

BTL: Bayesian threshold LASSO; GAW16: Genetic Analysis Workshop 16; GWAS: Genome-wide association studies; IRB: Institutional review board; LASSO: Least absolute shrinkage and selection operator; NARAC: North American Rheumatoid Arthritis Consortium; RA: Rheumatoid arthritis; SNP: Single-nucleotide polymorphism.

## Competing interests

The authors declare that they have no competing interests.

## Authors' contributions

OG-R participated in the design of the study and the statistical analyses and drafted the manuscript. EL participated in the design of the study and the statistical analyses, and helped revise the manuscript. ATV participated in the design of the study and helped revise the manuscript. CDE obtained IRB approval for the study, gained access to the dataset, participated in the design of the study, and helped revise the manuscript. KWB participated in the design of the study and helped revise the manuscript. All authors read and approved the final manuscript.
